# Loss of succinate dehydrogenase activity results in dependency on pyruvate carboxylation for cellular anabolism

**DOI:** 10.1038/ncomms9784

**Published:** 2015-11-02

**Authors:** Charlotte Lussey-Lepoutre, Kate E. R. Hollinshead, Christian Ludwig, Mélanie Menara, Aurélie Morin, Luis-Jaime Castro-Vega, Seth J. Parker, Maxime Janin, Cosimo Martinelli, Chris Ottolenghi, Christian Metallo, Anne-Paule Gimenez-Roqueplo, Judith Favier, Daniel A. Tennant

**Affiliations:** 1 INSERM, UMR970, Paris-Cardiovascular Research Center at HEGP, F-75015 Paris, France; 2 Université Paris Descartes, Sorbonne Paris Cité, Faculté de Médecine, F-75006 Paris, France; 3 Department of Genetics, Assistance Publique-Hôpitaux de Paris, Hôpital Européen Georges Pompidou, Paris, France; 4 Institute of Metabolism and Systems Research, College of Medical and Dental Sciences, University of Birmingham, Birmingham B15 2TT, UK; 5 Department of Bioengineering, University of California, San Diego, La Jolla, California 92093, USA; 6 Metabolic Biochemistry Laboratory, Hôpital Necker-Enfants Malades, F-75015 Paris, France; 7 INSERM, Unit 1124, F-75015 Paris, France

## Abstract

The tricarboxylic acid (TCA) cycle is a central metabolic pathway responsible for supplying reducing potential for oxidative phosphorylation and anabolic substrates for cell growth, repair and proliferation. As such it thought to be essential for cell proliferation and tissue homeostasis. However, since the initial report of an inactivating mutation in the TCA cycle enzyme complex, succinate dehydrogenase (SDH) in paraganglioma (PGL), it has become clear that some cells and tissues are not only able to survive with a truncated TCA cycle, but that they are also able of supporting proliferative phenotype observed in tumours. Here, we show that loss of SDH activity leads to changes in the metabolism of non-essential amino acids. In particular, we demonstrate that pyruvate carboxylase is essential to re-supply the depleted pool of aspartate in SDH-deficient cells. Our results demonstrate that the loss of SDH reduces the metabolic plasticity of cells, suggesting vulnerabilities that can be targeted therapeutically.

## Abstract (web summary)

Evidence suggests that the TCA cycle enzyme complex succinate dehydrogenase (SDH) may be dispensable for cell proliferation in some cancer cells. Here the authors show that SDH deficient cells become dependent on the mitochondrial enzyme pyruvate carboxylase for aspartate production and proliferation.

## Graphical abstract

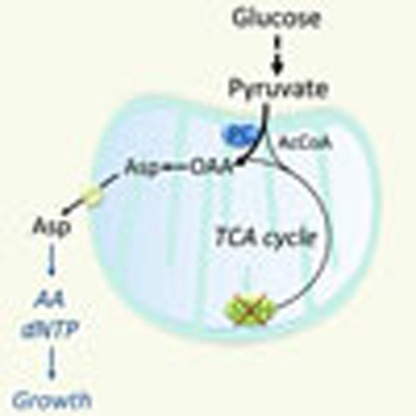

Pheochromocytoma (PCC) and paraganglioma (PGL) are neuroendocrine tumours arising in the adrenal medulla and in paraganglia of the autonomous nervous system, respectively. Since the original discovery of *SDHD* mutations in hereditary PGL in 2000, the genes encoding all four proteins that constitute the succinate dehydrogenase (SDH) complex as well as the required assembly factor (SDH assembly factor 2: *SDHAF2*) have been shown as tumour-suppressor genes in familial and apparently sporadic PCC and PGL^1^,^2^,^3^,^4^,^5^. Interestingly, *SDHB* mutation carriers were specifically shown to be predisposed to malignant^6^ and particularly aggressive forms of the disease^7^. The effects of all these mutations are to abolish SDH activity, which results in high steady-state intracellular concentrations of succinate^3^,^4^,^8^,^9^,^10^,^11^.

A growing number of tricarboxylic acid (TCA) cycle intermediates, namely succinate and fumarate, are now considered oncometabolites, acting in part through the aberrant activation of transcription factors and global epigenetic reprogramming^11^,^12^,^13^. Indeed, both SDH and fumarate hydratase-mutant tumours have been shown to elicit a pseudohypoxic phenotype through high steady-state levels of succinate and fumarate, respectively^11^,^14^,^15^,^16^. Tumours arising from such mutations demonstrate stabilization and activation of the hypoxia-inducible transcription factors, HIF1 and 2, which have been shown to elicit hypoxia-like alterations in metabolism, such as increased lactate dehydrogenase A (LDHA) expression and lactate production^8^,^9^,^17^,^18^. However, the direct metabolic consequences of SDH loss are as yet uncharacterized. It was recently shown that loss of another TCA cycle enzyme associated with a hereditary cancer syndrome, fumarate hydratase, led to significant metabolic reprogramming of the mitochondria, which was essential for continued cell viability^18^,^19^. Indeed, both genetic and hypoxia-mediated disruption of the TCA cycle has been suggested to result in alterations in cellular metabolic activity, resulting in cells that are more reliant on reductive carboxylation of glutamine for the provision of carbon for anabolic purposes than oxidative TCA cycle metabolism^20^,^21^,^22^. These data suggested that certain cell types are capable of re-wiring their metabolic network in response to the loss of a fully functional TCA cycle, but that despite this, the resulting metabolic phenotype is capable of sustaining oncogenic transformation.

We therefore sought to investigate how cells deficient in SDH activity through deletion of the SDHB subunit maintain their proliferation and viability. We found that in the absence of a fully functional TCA cycle, cells become deficient for a number of key central carbon metabolites, such as citrate, malate and aspartate. Aspartate synthesis is maintained through pyruvate carboxylation, which SDH-deficient cells become critically dependent on for their proliferation and survival. Finally, we found that, despite the use of reductive carboxylation to metabolize glutamine, SDH-deficient cells are unable to reverse their deficiency.

## Results

### Amino-acid metabolism is perturbed in SDH-mutated tumours

**Figure 1:**
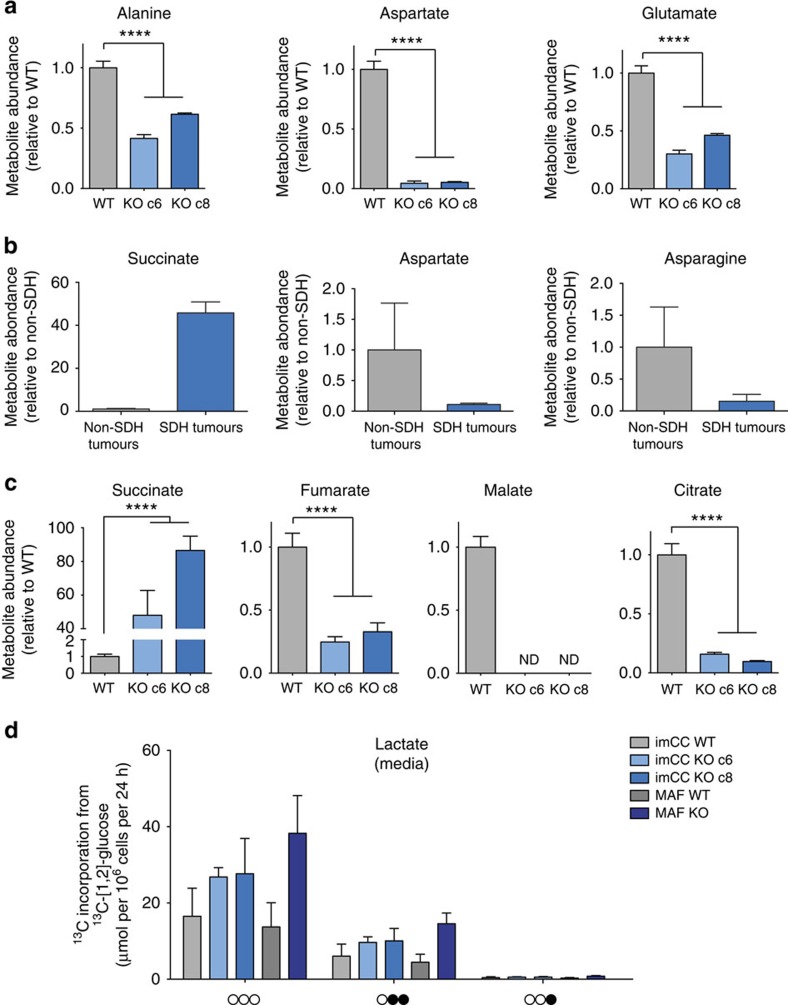
TCA cycle dysfunction in SDH-deficient cells. Loss of succinate dehydrogenase leads to perturbations in amino-acid metabolism, TCA cycle dysfunction and aerobic glycolysis. (**a**) Abundance of alanine, aspartate and glutamate decrease in imCC *Sdhb*-null (KO c6 and c8) cells relative to wild-type (WT) control, as assessed using GC–MS (*n*=3). ****P<0.0001 as calculated by a Kruskal–Wallis test. (**b**) Steady-state succinate, aspartate and asparagine in *SDH*-mutated patients’ tumours (*n*=3) compared with non-*SDH* mutated tumours (*n*=3). As expected, succinate is significantly increased, whereas aspartate and asparagine are decreased. (**c**) Abundance of succinate, fumarate, malate and citrate are significantly altered in *Sdhb*-KO imCC (c6 and c8) cells relative to WT control, as assessed using GC–MS (*n*=3). ****P<0.0001 as calculated by a Kruskal–Wallis test. (**d**) SDH-deficient cells exhibit aerobic glycolysis as shown by increase lactate production from glucose. After 24 h of incubation with medium containing ^13^C-[1,2]-glucose, the media were extracted and the abundance of different mass isotopomers of lactate excreted were quantified using NMR spectroscopy. ^13^C atoms (arising from glucose) are shown as filled circles, whereas ^12^C atoms are empty circles (*n*=3). All error bars shown represent s.d.

To explore predicted changes in tumourigenic metabolic pathways, we performed a bioinformatic analysis of the transcriptome of 186 PCC/PGL (including 23 *SDH*-mutated tumours) using a previously described subset of metabolic genes identified as being important in tumorigenesis^23^. This unbiased analysis segregated *SDH*-mutated from non-*SDH*-related tumours, suggesting that they exhibit a distinct metabolic phenotype (Supplementary Fig. 1 and Supplementary Table 1a,b). Gene ontology analysis of the differentially regulated pathways showed that the most highly dysregulated metabolic pathways involved amino-acid metabolism, with specific reference to the non-essential amino acids alanine, aspartate and glutamate as the most affected (Supplementary Table 1c,d). We performed an analysis of the steady-state concentrations of these amino acids using the previously characterized immortalized mouse chromaffin cell (imCC) lines (wild-type; WT, *Sdhb*^*−/−*^; clones 6 and 8) as a model, which represent the cell type from which PCC arise in patients. We found that all three amino acids identified from the transcriptomic screen were significantly altered in SDH-deficient cells, with aspartate concentrations particularly affected (Fig. 1a). In addition, steady-state concentrations of glycine, proline and serine were also altered, although the effect was not consistent between the clones with respect to glycine and proline (Supplementary Fig. 2a). To confirm the physiological significance of alterations in the concentrations of alanine, aspartate and glutamate, we performed a targeted analysis of amino-acid concentrations in *SDH*-mutated human PCC/PGL tumours compared with non-*SDH* mutated ones. Alongside succinate, which was greatly elevated as expected in *SDH*-mutated tumours, aspartate and the related amino acid, asparagine, were both decreased (Fig. 1b). In addition, the change in serine concentration was also recapitulated (Supplementary Fig. 2b).

### *Sdhb* ^*−/−*^ cells exhibit altered pyruvate metabolism

Aspartate production in most cells occurs mainly through oxidative TCA cycle metabolism, which SDH-deficient cells would be expected to lack. We therefore investigated the steady-state concentrations of TCA cycle intermediates, and observed, in addition to the previously reported truncated TCA cycle (high succinate, low fumarate and malate^11^,^16^), a significantly reduced steady-state citrate (Fig. 1c). Both reduced aspartate and citrate steady-state concentrations suggest alterations in the metabolism of the glycolytic metabolite, pyruvate, which also represents the glycolytic entry point into the TCA cycle. We investigated this phenomenon by incubating both the imCC cell model, and a novel *Sdhb*^*−/−*^ mouse adrenal fibroblast (MAF) cell line model (Supplementary Fig. 2c–e) with ^13^C-enriched glucose. Consistent with the pseudohypoxic phenotype previously reported in SDH-deficient cells^9^,^14^, we noted increased lactate production and excretion (Fig. 1d), along with the additional observation of increased alanine synthesis and excretion (Supplementary Fig. 3a).

**Figure 2:**
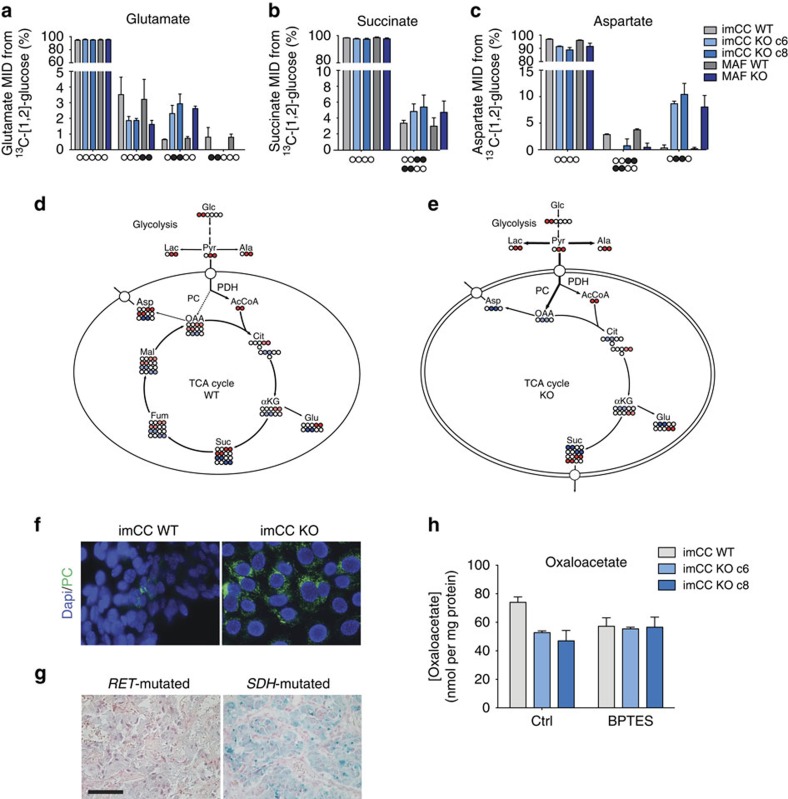
Loss of SDH-activity leads to increased pyruvate carboxylation. (**a**–**c**) Both imCC and MAF cell models were incubated for 24 h with ^13^C-[1,2]-glucose, and cell extracts subjected to NMR spectroscopy analysis of resulting incorporation of ^13^C into (**a**) glutamate, (**b**) succinate and (**c**) aspartate (*n*=3, error bars are s.d.). Reduction in the relative abundance of the ^13^C-[4,5]-glutamate and increase in ^13^C-[2,3]-glutamate isotopomers suggest increase in PC activity and relative decrease in PDH activity in SDH-deficient cells. This is confirmed by change in isotopomer distribution in aspartate towards the ^13^C-[2,3] isotopomer. ^13^C atoms are shown as filled circles, whereas ^12^C atoms are empty circles. (**d**,**e**) Diagrams describing the incorporation of carbons from glucose through PDH (red) or PC (blue) activity into (**d**) wild-type and (**e**) *Sdhb*^*−/−*^ (KO) cells. Abbreviations in metabolic diagrams: αKG; alpha-ketoglutarate, Ac, acetate; AcCoA, acetyl coenzyme A; Ala, alanine; Asp, aspartate; Cit, citrate; Fum, fumarate; Glc, glucose; Glu, glutamate; Lac, lactate; Mal, malate; OAA, oxaloacetate; PC, pyruvate carboxylase; PDH, pyruvate dehydrogenase; Pyr, pyruvate; Suc, succinate. (**f**) PC protein expression is increased in *Sdhb*^*−/−*^ (KO) cells compared with wild-type (WT) controls. (**g**) *SDH*-mutated PCC also shows significantly upregulated PC expression compared with non-SDH-mutated PCC harbouring a *RET* mutation. Scale bar, 50 μm. (**h**) OAA concentrations are decreased in *Sdhb*^*−/−*^ (KO c6 and c8) cells as measured using a citrate synthase-based enzymatic assay (*n*=3 with 3 replicates/experiment. Error bars are s.e.m.). The glutaminase inhibitor, BPTES, reduces OAA concentrations only in wild-type cells, showing the loss of dependency on glutaminase for OAA synthesis in SDH-deficient cells. MID, mass isotopomer distribution KO, Sdhb knockout.

Significant synthesis of metabolites arising downstream from pyruvate through the TCA cycle in both WT and *Sdhb* knockout (KO) cells were also observed through the detection of label incorporation from glucose into glutamate, succinate and aspartate (Fig. 2a–c). The isotopomers of glutamate and aspartate produced in the *Sdhb* WT and KO, however, were significantly different suggesting the implication of differential metabolic pathways (Fig. 2a,c, and Supplementary Fig. 3b). Mitochondrial pyruvate can either be oxidatively decarboxylated by pyruvate dehydrogenase (PDH) to form acetyl coA, or carboxylated by PC to form oxaloacetate (OAA). In WT cells, the ^13^C-[4,5]-glutamate species was the major isotopomer observed from ^13^C-[1,2]-glucose, consistent with the oxidative decarboxylation of pyruvate by PDH (Fig. 2a,d, and Supplementary Fig. 3b). However, in *Sdhb* KO cells, the relative contribution of the ^13^C-[4,5]-glutamate was reduced by about 50%, and replaced by ^13^C-[2,3]-glutamate, which represents incorporation of labelled OAA into the TCA cycle (Fig. 2a,e and Supplementary Fig. 3b). This suggests that in SDH-deficient cells there is an increase in OAA synthesis and metabolism through the activity of PC, which compensates for the reduction in pyruvate oxidation by providing necessary carbons for continued mitochondrial metabolism and cellular anabolism. Transamination of OAA produces aspartate, the metabolism of which we show to be dysregulated in SDH-deficient cells and tumours (Fig. 1a,b). We therefore examined ^13^C incorporation from glucose into this amino acid, and found that it confirmed the switch in pyruvate oxidation to carboxylation in the *Sdhb*^*−/−*^ cells. Indeed, although WT cells were observed to synthesize ^13^C-[1,2]/[3,4]-aspartate through oxidative TCA cycle activity, SDH-deficient cells showed a significant induction of PC activity to synthesize ^13^C-[2,3]-aspartate from mitochondrial OAA (Fig. 2c–e). Accordingly, expression of PC was induced in both the imCC cell line model (Fig. 2f), and in *SDH*-mutated tumours (Fig. 2g and Supplementary Table 2), which may support increased flux through this enzyme. As the sole source of mitochondrial OAA in SDH-deficient cells is through the activity of PC, we hypothesized that this metabolite may be limiting for the synthesis of citrate in SDH-deficient cells, resulting in the low steady-state citrate concentrations observed (Fig. 1c). Owing to the naturally low levels of OAA, we assessed steady-state concentrations using an enzymatic assay, and observed reduced OAA concentrations in SDH-deficient cells (Fig. 2h). Importantly, when cells were treated with the specific glutaminase inhibitor, Bis-2-(5-phenylacetamido-1,3,4-thiadiazol-2-yl)ethyl sulphide (BPTES), concentrations of OAA were decreased only in the WT cells, showing as expected, that SDH-deficient cells do not metabolize glutamate produced by glutaminase as a source of OAA. Interestingly, we noted enrichment of isotope incorporation from glucose into acetate in both cells and media, a ketone body product of acetyl-coA in SDH-deficient cells (Supplementary Fig. 3d,e), suggesting that *Sdhb*-deficient cells and perhaps tumours may exhibit a ketogenic phenotype.

### Aspartate synthesis is PC-dependent in *Sdhb* ^*−/−*^ cells

**Figure 3:**
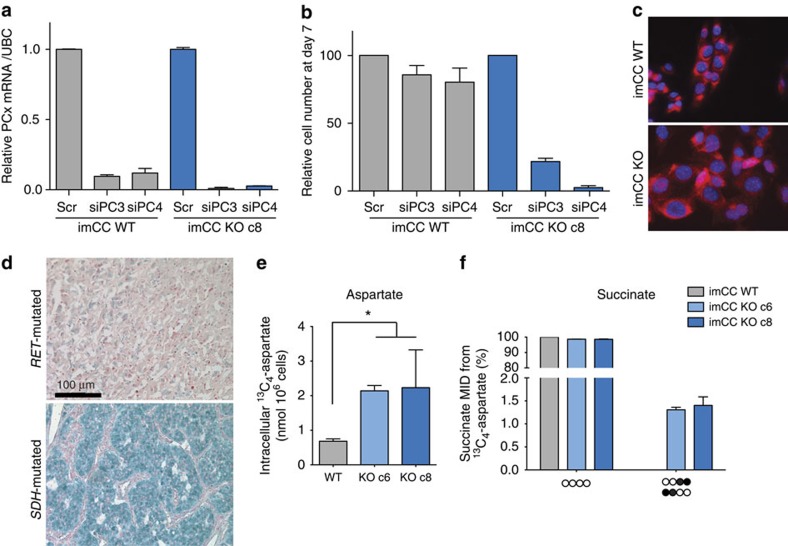
Mitochondrial aspartate synthesis by PC is essential for *Sdhb*^*−/−*^ cell proliferation and survival. (**a**) Successful knockdown of PC expression in both wild-type (WT) and *Sdhb*^*−/−*^ cells assessed by quantitative RT–PCR (*n*=3, error bars are s.e.m), results in (**b**) inhibition of proliferation and decrease in survival only in *Sdhb*^*−/−*^ (KO c8) cells, whereas scrambled (scr) siRNA shows no effect (*n*=3, error bars are s.e.m). (**c**,**d**) Glutamate/aspartate antiporter SLC25A13 protein expression in (**d**) *Sdhb*^*−/−*^ cells and (**e**) *SDHB*-mutated PCC consistent with increased requirement for export of aspartate from the mitochondria. Scale bar, 100 μm. (**e**) *Sdhb*^*−/−*^ imCC (KO c6 and c8) demonstrate increased uptake of aspartate when incubated with 0.1 mM ^13^C_4_-aspartate for 48 h compared with wild-type cells, consistent with an increased requirement for aspartate in SDH-deficient cells due to dysfunctional synthesis (*n*=3, error bars are s.d.). *P=0.05, Kruskal–Wallis test. (**f**) Labelled aspartate is metabolized more rapidly into cellular metabolites such as succinate in *Sdhb*^*−/−*^ cells (*n*=3, error bars are s.d.). MID, mass isotopomer distribution KO, Sdhb knockout.

The increased reliance of SDH-deficient cells on PC activity for aspartate synthesis, and the central role of aspartate in cellular anabolism suggested that loss of PC activity would be incompatible with cell growth and proliferation. We therefore tested whether SDH-deficient cells were dependent on PC activity by knocking down its expression in the imCC cell model using two independent short interfering RNA (siRNA) sequences (Fig. 3a and Supplementary Fig. 4a). PC deficiency resulted in little effect on cell number in WT cells, but resulted in complete ablation of proliferation in SDH-deficient cells (Fig. 3b and Supplementary Fig. 4b). This, hence, confirmed the essential role of PC in SDH-deficient cell growth and survival.

To be available for cytosolic biosynthetic processes, aspartate produced in the mitochondria is exported across the mitochondrial inner membrane against a gradient of glutamate using an aspartate-glutamate antiporter. Although expression of neither of the two antiporters, SLC25A12 and 13, was increased in SDH-deficient cells (Fig. 3c and Supplementary 4c and d), protein expression of SLC25A13 was noted as significantly upregulated in SDH-deficient tumours (Fig. 3d and Supplementary Table 2). This supported the model in which SDH-deficient cells may be more dependent on the production of aspartate from PC-derived OAA in the mitochondria, and may therefore require increased activity of the appropriate transporters to export it for biosynthetic metabolism in the cytosol. As our cell line models were grown in DMEM, which lacks aspartate, the cells are entirely reliant on endogenous synthesis of aspartate for their growth and proliferation. We hypothesized that, if available, SDH-deficient cells would take up more exogenous aspartate than their WT counterparts. We therefore supplemented both WT and KO cells with exogenous ^13^C_4_-aspartate and found that cells lacking SDH activity demonstrated increased uptake and metabolism of this amino acid (Fig. 3e,f and Supplementary Fig. 4e).

### SDH-deficiency elicits reductive carboxylation of glutamine

**Figure 4:**
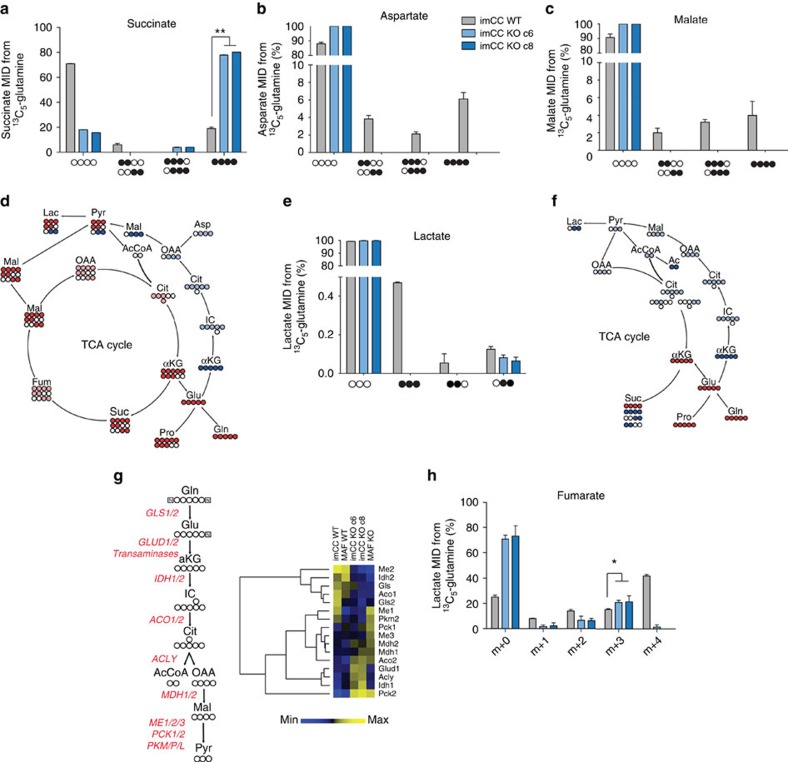
*Sdhb*^*−/−*^ cells exhibit dysfunctional glutamate metabolism leading to reductive carboxylation. Glutaminolysis leads to incorporation of ^13^C_5_-glutamine into (**a**) succinate, (**b**) aspartate and (**c**) malate in wild-type cells. (**a**) A significantly greater proportion of the succinate pool in *Sdhb*^*−/−*^ cells directly arises from glutamine incorporation into proximal TCA cycle metabolites (**P=0.0036, Kruskall–Wallis performed on fully labelled values). However, no isotopomers of aspartate (**b**) or malate (**c**) were observed in *Sdhb*^*−/−*^ cells. (**d**) Diagrammatic representation of the oxidative (red) and reductive (blue) metabolism of SDH wild-type cells. (**e**) Mass isotopomers of lactate after incubation of cells with ^13^C_5_-glutamine suggest incorporation of carbons into lactate from reductive metabolism in SDH-deficient cells. As the ^13^C-[1,2]-lactate and ^13^C-[2,3]-lactate isotopologues are made in equal ratios through the action of oxidative TCA cycle metabolism in wild-type cells, the ^13^C-[2,3]-lactate isotopomer in these cells is an over-estimation of reductive carbon incorporation. (**f**) Diagrammatic representation of the oxidative (red) and reductive (blue) metabolism of *Sdhb*^*−/−*^ cells. (**g**) The enzymes involved in the reductive metabolism of glutamine are differentially expressed in SDH-deficient cells. Of interest, most enzymes with cytosolic reductive activity are upregulated, whereas most with mitochondrial-localized reductive activity are downregulated. (**h**) Mass isotopomer distribution (MID) of intracellular fumarate after cells were incubated for 48 h with ^13^C_5_-glutamine as measured using GC–MS. SDH-deficient cells show decreased isotopomers produced through oxidative metabolism (for example, m+1, m+2 and m+4), whereas instead showing increased ^13^C incorporation through reductive metabolism (m+3; *P*=0.05, Kruskal–Wallis test on m+3 isotopomer values only). Abbreviations in metabolic diagrams: αKG, alpha-ketoglutarate; Ac, acetate; AcCoA, acetyl coenzyme A; Ala, alanine; Asp, aspartate; Cit, citrate; Fum, fumarate; Gln, glutamine; Glu, glutamate; IC, isocitrate; Lac, lactate; Mal, malate; OAA, oxaloacetate; Pro, proline; Pyr, pyruvate; Suc, succinate. KO, Sdhb knockout. For all metabolic data (**a**,**b**,**c**,**e** and **h**), *n*=3 and error bars are s.d.

Having shown that SDH-deficient cells display significantly increased synthesis of aspartate from glucose using PC activity, and that they uptake more exogenous aspartate, we sought to investigate whether glutamine metabolism was able to at least partially compensate for the putative deficiency in cytosolic aspartate. Glutamine is not only an amino acid, but also a major source of carbon, the availability of which is essential for *de novo* nucleotide biosynthesis and important for the synthesis of almost all non-essential amino acids. We incubated both WT and *Sdhb*-deficient imCCs with ^13^C_5_-glutamine and the resulting polar extracts of cells and media were analysed for the incorporation of ^13^C into metabolites. *Sdhb* KO cells were found to incorporate significantly more glutamine carbon into succinate than their WT counterparts (Fig. 4a), suggesting enhanced flux of glutamine carbons into the TCA cycle compared with glucose. This flux of glutamine carbons into the mitochondria could be used to support export of aspartate synthesized through PC activity in order to support cellular anabolism through the aspartate/glutamate antiporter (SLC25A12 and 13).

Carbon from glutamine was significantly enriched in both aspartate and malate in positions consistent with oxidative TCA cycle activity in WT imCC (^13^C_4_, ^13^C-[1,2] and ^13^C-[3,4]-malate and aspartate; Fig. 4b–d). As expected, SDH-deficient cells produced neither isotopomers of these metabolites because of their truncated TCA cycle (Fig. 4b,c,f). ^13^C-[1,2,3]/[2,3,4]-malate and aspartate isotopomers were also noted in WT cells, but not in *Sdhb* KO clones (Fig. 4b,c). These isotopomers are likely products of the reductive carboxylation of glutamine—an observation that is supported by the isotopomers of lactate identified in the same samples, which can be produced through the activity of malic enzyme or phosphoenolpyruvate carboxykinase, both of which have been previously reported in tumours^20^,^24^,^25^ (Fig. 4e and Supplementary Fig. 4f). Interestingly, although label incorporation was observed in neither malate nor aspartate in SDH-deficient cells, evidence of reductive carboxylation of glutamine could be observed from the only isotopomer of lactate identified in these cells and in the media (^13^C-[2,3], Fig. 4e,f and Supplementary Fig. 4f). This suggests that, in common with other systems in which the oxidative TCA cycle is perturbed, there may be an increased reliance on reductive carboxylation for the supply of carbons to metabolic intermediates to support cellular anabolism^20^,^21^,^22^. This is likely to be supported by the concerted, supportive transcriptional response observed (Fig. 4g). In case the absence of observable label incorporation into aspartate and malate in the KO cells was due to the sensitivity of our nuclear magnetic resonance (NMR) spectroscopy approach, we also used a longer incubation time (72 h) to analyse incorporation of glutamine carbons into these metabolites, and used gas chromatography-mass spectrometry (GC–MS) to analyse the resulting extracts. These data confirmed our previous analysis (Supplementary Fig. 4g–h), and in addition we observed increased proportions of the m+3 isotopomer of fumarate, consistent with reductive carboxylation (Fig. 4h). However, the very low steady-state concentration of malate and aspartate found in these cells (Fig. 1a,c) did not allow for quantification ^13^C incorporation from glutamine. This suggests that although these two metabolites are produced from glutamine, their metabolism is rapid enough to retain very low steady-state concentrations in SDH-deficient cells.

## Discussion

Metabolic reprogramming has been proposed to be a core hallmark of cancer^26^. As a central metabolic hub, it is likely that some aspect of mitochondrial metabolic reprogramming exists in most tumours^27^. Originating from a defect in a central mitochondrial enzyme complex, *SDH*-mutated tumours constitute an ideal genetic model for deciphering how metabolic adaptation fuels cell growth and proliferation. It has long been known that SDH-deficient PCC/PGL demonstrate an altered metabolic phenotype: not only through the inactivation of SDH mitochondria function *per se*, but also via a pseudohypoxic phenotype mediated by the effect of succinate accumulation on the cellular transcriptome^11^,^12^,^16^. However, the details of the direct effects of metabolic transformation due to loss of SDH activity were as yet uncharacterized. Our transcriptomic and metabolic tracer studies demonstrate that amino-acid metabolism is altered in SDH-deficient cells as a result of re-wiring of mitochondrial metabolic pathways. We demonstrate that SDH-deficient cells increase PC-dependent carboxylation of pyruvate to synthesize OAA in order to produce aspartate as well as continue a truncated oxidative TCA cycle in the mitochondria. In addition, SDH-deficient cells are highly dependent upon mitochondrial aspartate production from OAA, as disruption of synthesis resulted in complete ablation of proliferation and a loss of viability. Aspartate constitutes a key metabolic hub in the cell, being a major precursor for protein and nucleotide biosynthesis, as well as critical for the synthesis of other ‘non-essential’ amino acids such as arginine and asparagine. With limited means of producing aspartate in SDH-deficient cells, the significant number of metabolic pathways that depend on it are likely to be unable to cope with a decline in supply, as demonstrated here. This loss of metabolic plasticity means that changes in the rate of synthesis of aspartate is likely to determine the anabolic and proliferative state of the cell. Finally, we show that glutamine and glutamate are used to provide metabolic intermediates that are lacking because of the truncation of the TCA cycle, but that the flux appears to be insufficient to fully compensate for the lack of oxidative TCA cycle activity. Altogether, our data indicate a significant loss of metabolic plasticity in SDH-deficient cells, and suggest novel therapeutic targets that would specifically disrupt the metabolism of these tumours.

## Methods

### Tumour samples

Tumours were collected prospectively by the French ‘Cortico et Médullosurrénale: les Tumeurs Endocrines’ network. Ethical approval for the study was obtained from the institutional review board (Comité de Protection des Personnes Ile de France III, June 2012). Written informed consent for the sample collection and subsequent analyses was obtained from all patients. The procedures used for PCC/PGL diagnosis were in accordance with both internal and international clinical practice guidelines^28^,^29^. Diagnosis was confirmed by histology in every case. A total of 202 consecutive cases of PCC/PGL, recruited over 15 years (1993–2008), were included in this study. Fresh tumour samples collected during surgery were immediately frozen and stored in liquid nitrogen until processing. Formalin-fixed paraffin-embedded tissues were used for immunohistochemical analyses.

### Generation of MAF cell lines

Primary MAF cell cultures were obtained from entire adrenal glands of 6-week-old *Sdhb*^*lox/lox*^ mice^12^. Briefly, glands were dissected and placed into Ca^2+^-and Mg^2+^-free Locke’s buffer (154 mM NaCl, 2.6 mM KCl, 2.2 mM K_2_HPO_4_, 0.95 mM KH_2_PO_4_, 10 mM glucose). Glands were digested 30 min in digestion buffer (glutamate- and pyruvate-free DMEM, 5.5 mM cysteine-HCl, 1 mM CaCl_2_, 0.5 mM EDTA) containing 0.22-μm-filtered papain (40 U ml^−1^; Worthington) at 37 °C and cells placed in culture. *Sdhb* knockout was achieved after infection with 107 PFU ml^−1^ of an adenovirus expressing Cre-recombinase (Ad5CMV-Cre, CellBioLabs). All studies were performed in accordance with the relevant guidelines of the French Ministry of Agriculture (Authorization Executive Order A751532) for scientific experimentation on animals, European Communities Council Directive and international ethical standards. Cells were infected with 10^7^ PFU ml^−1^ of an adenovirus expressing Cre-recombinase (Ad5CMV-Cre, CellBioLabs). A clonal approach by limiting dilution cloning assay was used to obtain homogenous *Sdhb*^*−/−*^ (KO) and *Sdhb*^*lox/lox*^ (referred to as WT) MAF clones.

### Cell culture

ImCC^12^ and MAF were cultured in DMEM, high glucose, GlutaMAX supplement, pyruvate medium (GIBCO) supplemented with 10% fetal bovine serum (GIBCO) and maintained in a humidified 5% CO_2_ atmosphere at 37 °C. Mycoplasma contamination was ruled out using the PCR Mycoplasma Test kit I/C (PromKine, PK-CA91-1048).

### Gene expression data

Gene expression profiles of human tumours were available from a previous study, in which 186 PCC/PGL were analysed^30^. All the samples were genotyped for the presence or the absence of germline and somatic mutations in well-known PCC/PGL susceptibility genes. In total, there are 23 *SDHx*, 38 *VHL*, 30 *NF1*, 14 *RET*, 1 *TMEM127*, 3 *MAX*, 6 *HRAS*, 3 *MET*, 65 sporadic, 1 *NF1/MET*, 1 *RET/HRAS* and 1 *TP53/CDKN2A/MET*^31^. HG-U133 Plus 2.0 Affymetrix GeneChip data from this previous study are available online as ArrayExpress entry E-MTAB-733 (http://www.ebi.ac.uk/arrayexpress/).

Gene expression profiles of WT and *Sdhb*^*−/−*^ MAF, imCC c6 and imCC c8 were assessed in duplicates using the GeneChip Mouse Gene 1.0 ST Array. Data are available as ArrayExpress entry E-MTAB-3403.

### ^13^C-glucose, ^13^C-aspartate and ^13^C-glutamine labelling

Cells (6 × 10^6^) were plated onto two 15-cm dishes and cultured in standard medium for 24 h. Cells were washed in PBS and the medium was then replaced by either fresh DMEM (no glucose) with 1 mM sodium pyruvate and 10 mM ^13^C_2_-[1,2]-D-glucose (Sigma) or DMEM (high glucose with pyruvate, no glutamine) supplemented with 2 mM ^13^C_5_-L-glutamine (Sigma) for 24 h. For aspartate labelling, cells were cultured in standard medium supplemented with 0.1 mM ^13^C_4_-L-aspartate for 48 h.

### Extraction of polar metabolites and NMR spectroscopy

Cells were washed with ice-cold PBS and scraped in a pre-cooled acetonitrile/methanol/water (55:35:10) solution. The cell lysates were vortexed for 15 min at 4 °C and immediately centrifuged at 15,000*g* for 15 min at 0 °C. The supernatants were finally evaporated using a SpeedVac concentrator and stored at −80 °C until further analyses. For the metabolomic extraction of conditioned media, 600 μl of an acetonitrile/methanol (60:40) solution was added to 200 μl of cell media, and then processed as described above. Dried NMR samples were resuspended in 60 μl of 100 mM sodium phosphate buffer containing 500 μM TMSP ((3-trimethylsilyl)propionic-(2,2,3,3-d4)-acid sodium salt) and 10% D_2_O, pH 7.0. Samples were vortexed, sonicated (5–15 min) and centrifuged briefly (x2), before transferred to 1.7 mm NMR tubes using an automatic Gilson. Extraction was performed on three different cultures for each labelling experiments.

One-dimensional (1D)-^1^H-NMR spectra and two-dimensional (2D)-^1^H,^13^C-Heteronuclear Single Quantum Coherence Spectroscopy (HSQC) NMR spectra were acquired using a 600-MHz Bruker Avance III spectrometer (Bruker Biospin) with a TCI 1.7 mm z-PFG cryogenic probe at 300 K. Spectral widths were set to 7,812.5 and 24,155 Hz for the ^1^H and ^13^C dimensions, respectively. 16,384 complex data points were acquired for the 1D-spectra and 512 complex data points were acquired for the ^1^H dimension of 2D-^1^H,^13^C-HSQC NMR spectra. An exponentially weighted non-uniform sampling scheme was used for the indirect dimension. Here, 30% of 8,192 complex data points (2,458) were acquired. 128 transients were recorded for the 1D-NMR spectra with a relaxation delay of 4 s, and two transients were recorded for the 2D-^1^H,^13^C-HSQC NMR spectra with a relaxation delay of 1.5 s. Each sample was automatically tuned, matched and then shimmed (1D-TopShim) to a TMSP line width of <2 Hz before acquisition of the first spectrum. Total experiment time was ∼15 min per sample for 1D-^1^H-NMR spectra and 4.5 h per sample for 2D-^1^H,^13^C-HSQC NMR spectra. 1D-^1^H-NMR spectra were processed using the MATLAB-based MetaboLab software^32^. All 1D data sets were zero-filled to 131,072 data points before Fourier Transformation. The chemical shift was calibrated by referencing the TMSP signal to 0 p.p.m. 1D-spectra were manually phase corrected. Baseline correction was achieved using a spline function^32^. 1D-^1^H-NMR spectra were exported into Bruker format for metabolite identification and concentration determination using Chenomx 7.0 (ChenomxINC). 2D-^1^H,^13^C-HSQC NMR spectra were reconstructed using compressed sensing in the MDDNMR and NMRpipe software^33^,^34^,^35^. The final spectrum size was 1,024 real data points for the ^1^H dimension and 16,384 real data points for the ^13^C dimension. Analysis was performed using MetaboLab and pyGamma software was used in multiplet simulations^36^. The methyl group of lactate was used to calibrate the chemical shift based on its assignment in the human metabolome database^37^.

### Metabolite extraction and GC–MS analysis

Cells were seeded onto six-well plates and cultured in standard medium for 24 h. Media was then replaced by DMEM (high glucose with pyruvate, no glutamine) supplemented with 2 mM ^13^C_5_-L-glutamine (Sigma) for 72 h. At the conclusion of tracer experiments, culture media were aspirated and cells were washed twice with 0.9% NaCl saline solution. Methanol (−80 °C) and water, containing 1 μg per well norvaline as internal standard (4 °C), were immediately added to each well and cells were scraped and transferred to tube. Chloroform (−20 °C) was added to each tube followed by vortex and separation into polar and nonpolar fractions by centrifugation. The polar fractions were transferred to new tubes and evaporated to dryness by SpeedVac concentration.

Derivatization of polar metabolites has been previously described^20^,^38^. Briefly, polar metabolites were derivatized to form methoxine-TBDMS derivatives by incubation with 2% methoxylamine hydrochloride dissolved in pyridine at 37 °C for 1 h followed by addition of *N*-tert-butyldimethylsilyl-*N*-methyltrifluoroacetamide with 1% tert-butyldimethylchlorosilane incubated at 37 °C for 30–60 min. Derivatized polar samples were analysed by GC–MS using a DB-35MS column (30 m × 0.25 mm i.d. × 0.25 μm) installed in an Agilent 7890B GC interfaced with an Agilent 5977A MS. Mass isotopomer distributions were determined by integrating metabolite ion fragments and corrected for natural abundance as previously described^39^.

Metabolite ion counts were normalized to norvaline and isoleucine ion counts to normalize for varying derivatization efficiency and cell number, respectively.

### siRNA experiments

A total of 50,000 cells were plated onto 12-well plates in standard culture conditions. Twenty-four hours later, they were transfected with 150 ng of Silencer Select Pre-designed siRNA against PC (siRNA references s71354 and s71355; Life Technologies) or Silencer Select negative control using RNAiMax reagent (Life Technologies) as described by the manufacturer. Forty-eight hours post transfection, cells were passaged and efficiency of gene extinction was evaluated by immunofluorescence and reverse transcription–quantitative PCR (qPCR). Passaged cells were retransfected 24 h later to maintain long-term extinction.

### Reverse transcription–qPCR

RNA extractions were performed with the miRNeasy kit (Qiagen). A total of 500 ng RNA per sample was used for reverse transcriptions using the RT reactions iScript cDNA Synthesis Kit (Bio-Rad). qPCR was performed with 10 ng of cDNA preparation and the iTaq Universal SYBR Green Supermix (Bio-Rad) in a CFX96 Real-Time machine (Bio-Rad). The expression of PC in each sample was normalized against the expression of Ubiquitin C used as housekeeping gene. The primer sequences and qPCR conditions are available upon request. The expression of PC in cells transfected with siRNA relative to the one in cells transfected with scrambled control was calculated with the 2-ΔΔCT method.

### Immunofluorescence

Cells plated on glass coverslips (Thermo scientific) were washed in PBS and fixed for 20 min in ice-cold paraformaldehyde 4% and permeabilized/blocked in 1% BSA, 0.1% Triton for 30 min. Primary antibody (anti-PCB antibody (3H2AD9; Abcam, ab110314), 1:100) was incubated overnight at 4 °C. Secondary antibodies (Alexa Fluor 488 Goat Anti-Mouse IgG (1/2,000, Invitrogen) were incubated for 1 h, at room temperature in the dark. Slides were mounted in Vectashield, containing DAPI and acquisitions were performed using Axioimager Z1 Zeiss, with apotome system.

### Cell proliferation

A total of 50,000 cells were plated on six-well plates, transfected as described and passed before reaching confluence into 10 cm dishes. Growth curves were established by counting cells every 48 h after transfection for 8 days. Experiments were performed three times, in duplicates.

### Organic acid assays

Six snap-frozen tumour samples (1 *SDHD*, 2 *SDHB*, 2 *NF1* and 1 *MAX*-mutated) and six imCC samples (3 WT and 3 KO) were processed by organic extraction with ethylacetate, derivatization with *N*,*O*-bis(trimethylsilyl) trifluoroacetamide with 1% trimethylchlorosilane, and analysed by gas chromatography-tandem mass spectrometry on a GC–MS triple quadrupole (Scion TQ, Brüker Daltonics). Analytes were identified according to retention time and mass spectrum in selected reaction monitoring mode based on standard spectral reference libraries.

### Immunohistochemistry

Six-micrometre sections were cut from 27 paraffin-embedded tumour samples (Supplementary Table 2) and mounted on Superfrost plus slides. The following antibodies and a standard procedure were used for immunohistochemistry^40^ with Histogreen as a chromogen: anti-PC (1:100, ab115579, abcam), anti-SCL25A13 (1:50, abcam). Antigen retrieval was performed by boiling slides in Tris-EDTA buffer (pH9) for 45 min for PC and in citrate buffer (pH6) for SLC25A13. Images were acquired with a Leica DM400B microscope with Leica Application Suite software version 2.8.1, and a Leica DFC420C camera (Leica).

## Additional information

**Accession code:** Gene expression profiles of wild-type and Sdhb–/– MAF, imCC c6 and imCC c8 are available at ArrayExpress with entry E-MTAB-3403.

**How to cite this article:** Lussey-Lepoutre, C. *et al*. Loss of succinate dehydrogenase activity results in dependency on pyruvate carboxylation for cellular anabolism. *Nat. Commun.* 6:8784 doi: 10.1038/ncomms9784 (2015).

## Supplementary Material

Supplementary Information Supplementary Figures 1-4 and Supplementary Table 2

Supplementary Data 1 Supplementary Table 1
