# Supplementary material for: Loss of succinate dehydrogenase activity results in dependency on pyruvate carboxylation for cellular anabolism
Source: Nat Commun. 2015 Nov 2;6:8784. doi: 10.1038/ncomms9784 (PMC4632646; doi:10.1038/ncomms9784)
Supplement: Supplementary Information — Supplementary Figures 1-4 and Supplementary Table 2 [file ncomms9784-s1.pdf]

Loss of succinate dehydrogenase activity results in dependency on pyruvate carboxylation for cellular anabolism

Charlotte Lussey-Lepoutre, Kate ER Hollinshead, Christian Ludwig, Mélanie Menara, Aurélie Morin, Luis-Jaime Castro-Vega, Seth J Parker, Maxime Janin, Cosimo Martinelli, Chris Ottolenghi, Christian Metallo, Anne-Paule Gimenez-Roqueplo, Judith Favier and Daniel A Tennant.

List of supplementary Figures and Tables

- Supplementary Figure 1
- Supplementary Figure 2
- Supplementary Figure 3
- Supplementary Figure 4
- Supplementary Table 1 (see data sheet)
- Supplementary Table 2

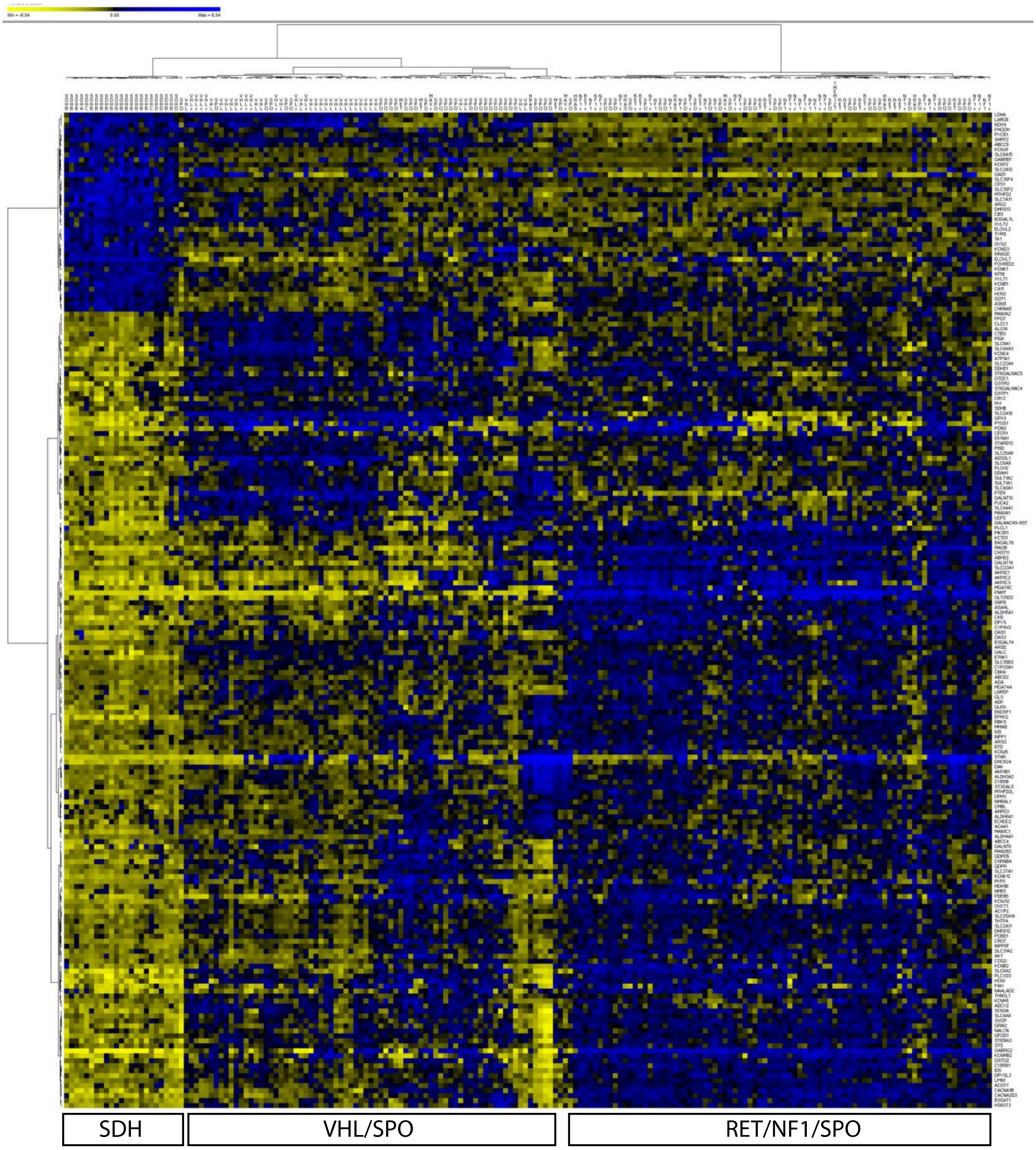

Supplementary Figure 1: Transcriptome of cancer-associated metabolic genes shows altered metabolic profile of SDH-mutated tumours. Gene expression data from 186 PCC/PGL that were profiled on the HG-U133 Plus 2.0 Affymetrix GeneChip were evaluated for a list of 2556 genes that encode for metabolic enzymes and transporters, adapted from the list reported by Possemato *et al*. 581 genes were differentially expressed (adjusted p value <0.01; Bonferroni multiple test comparisons): 300 upregulated and 281 downregulated in SDH-mutated tumours vs SDH WT mutated tumours. Heatmap shows classification using the 200 differentially expressed genes (40 upregulated and 160 downregulated) with a fold change >1.7.

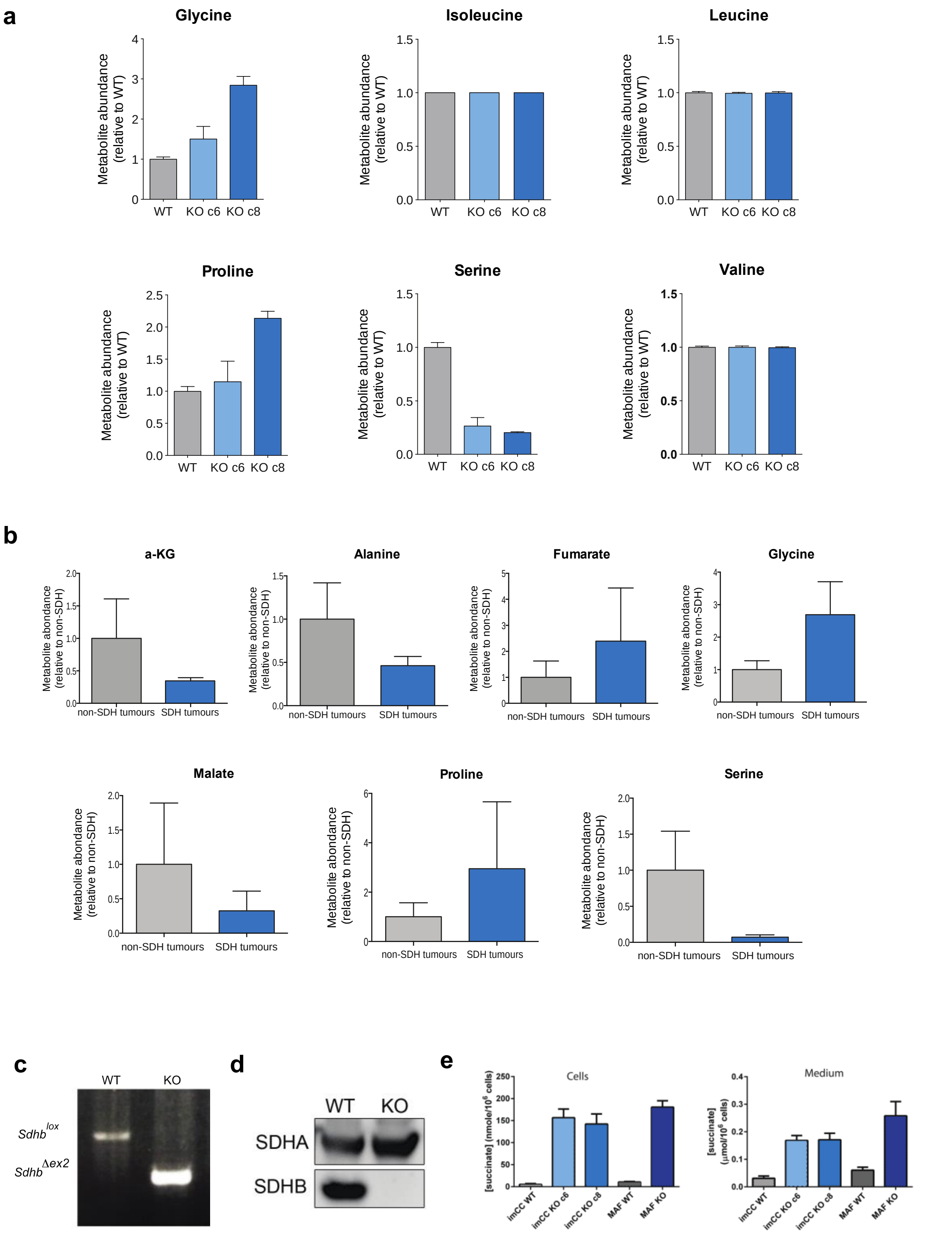

Supplementary Figure 2

a) Abundance of glycine, isoleucine leucine, proline, serine and valine in imCC *Sdhb*-null (KO c6 and c8) cells relative to wild-type (WT) control, as assessed using GC-MS (n=3, mean +/- SD). Serine is significantly decreased, while glycine and proline are increased, only in clone 8 **b)** Steady-state αKG, alanine, fumarate, glycine, malate, proline and serine in SDH-mutated patient tumours (n=3) compared to SDH WT tumours (n=3). Data are mean +/- SD. As in KO cells, serine is significantly decreased, in SDH-mutated tumours. Data are mean+/-SD c**)** genotyping of the mouse adrenal fibroblast *Sdhb* knockout cell line (MAF KO) shows homozygous deletion of Sdhb exon 2 (Δex2) resulting in **d)** complete loss of SDHB protein expression. **e)** Steady-state concentrations of succinate are increased in cells and in the medium from SDH-deficient imCC and MAF (n=3, mean +/- SD).

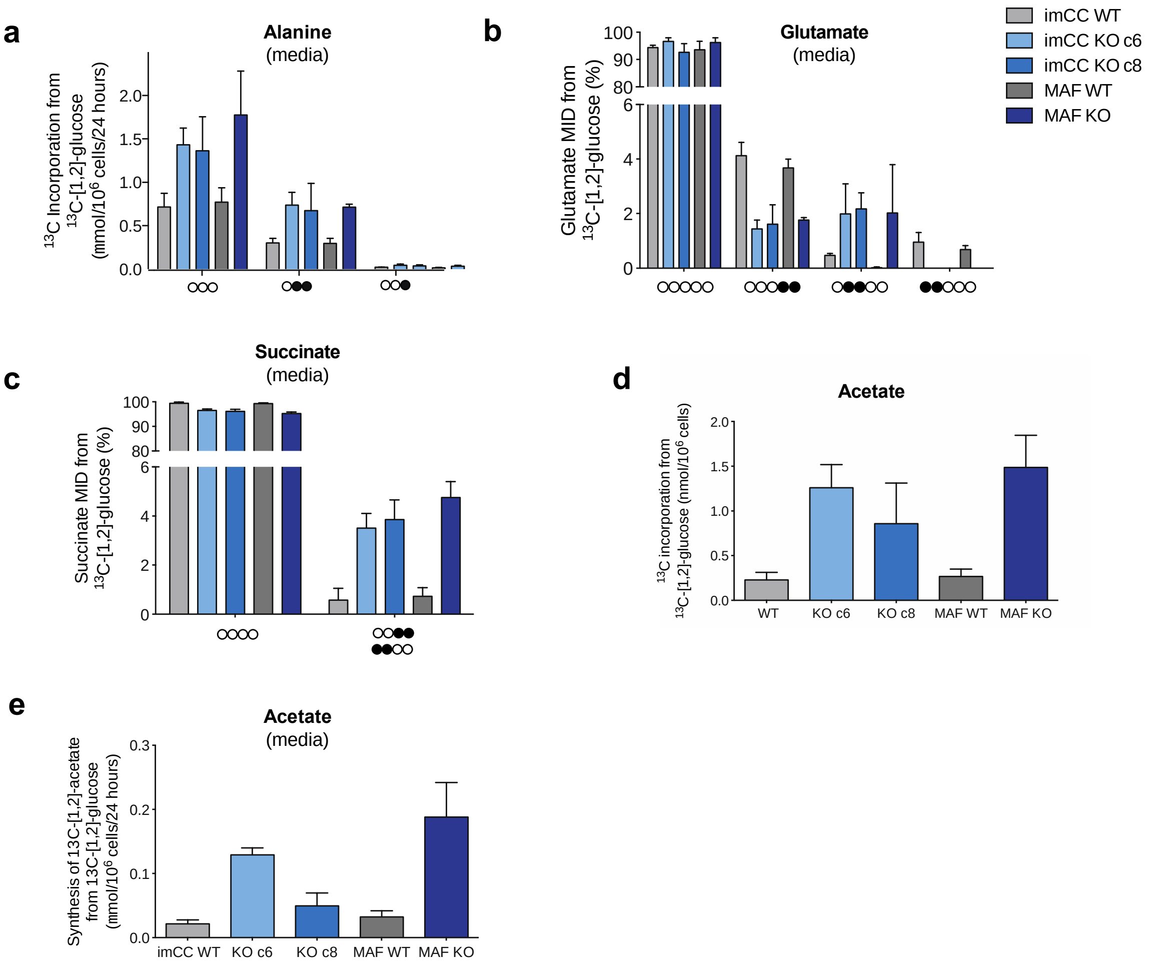

Supplementary Figure 3

a) SDH-deficient imCC and MAF exhibit increased alanine production and excretion. After 24h of incubation with medium containing ^{13}C-[1,2]-glucose, media was extracted and the abundance of different mass isotopomers of alanine excreted were quantified using NMR spectroscopy. ^{13}C atoms (arising from glucose) are shown as filled circles, while ^{12}C atoms are empty circles. Incorporation of ^{13}C into **b)** glutamate and **c)** succinate. Reduction in the relative abundance of the ^{13}C-[4,5]-glutamate and increase in ^{13}C-[2,3]-glutamate isotopomers suggests increase in PC activity and relative decrease in PDH activity in SDH-deficient cells. ^{13}C atoms are shown as filled circles, while ^{12}C atoms are empty circles. MID: mass isotopomer distribution. **d-e)** SDH-deficient cells produce and excrete significantly more acetate from glucose than wild-type cells. n=3 replicates, data indicate mean +/- SD.

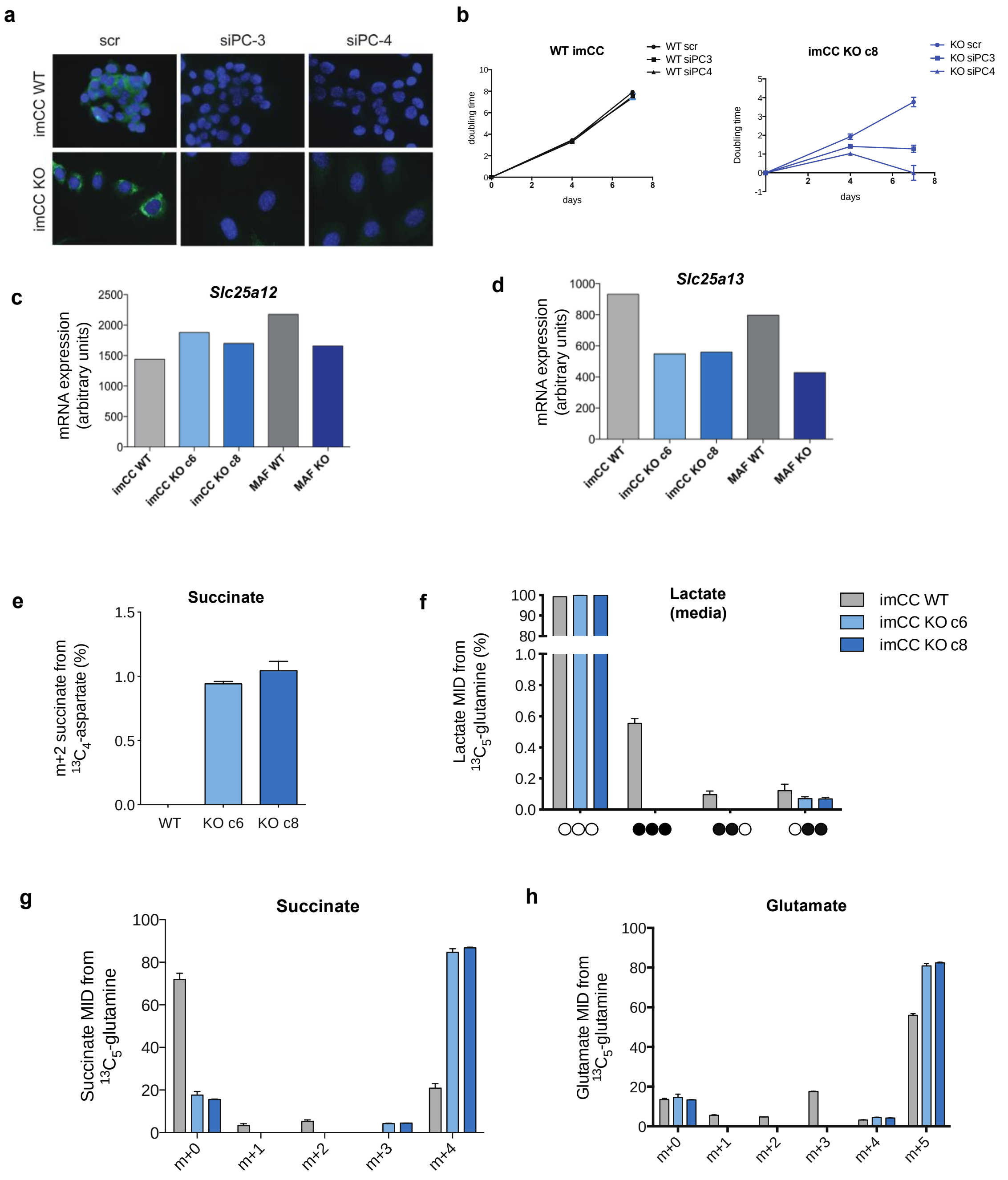

Supplementary Figure 4

a) Reduction in the expression of PC using siPC sequences #3 and #4 results **b)** in a significant reduction doubling time calculated over 7 days in SDH-deficient cells (right panel) while proliferation of wild-type cells (left panel) is unaffected. Data are mean +/- SEM (n=3). mRNA expression of **c)** *Slc25a12* and **d)** *Slc25a13* evaluated by microarray in WT and SDH-deficient cells **e)** Amount of m+2 label (%) from ^{13}C4-asparate incorporated into succinate was measured using GC-MS (n=3, data are mean +/- SD). **f)** Mass isotopomer distribution (MID) of lactate in media after incubation of cells with $^{13}C_5$-glutamine suggest incorporation of carbons into lactate from reductive metabolism in SDH-deficient cells. As the ^{13}C-[1,2]-lactate and ^{13}C-[2,3]-lactate isotopologues are made in equal ratios through the action of oxidative TCA cycle metabolism in wild-type cells, the ^{13}C-[2,3]-lactate isotopomer in these cells is an over-estimation of reductive carbon incorporation (n=3, data are mean +/- SD). **g-h)** MID of intracellular succinate and glutamate after cells were incubated for 72 hours with $^{13}C_5$-glutamine as measured using GC-MS (n=3, data are mean +/- SD).

Legend to Supplementary Table 1- Transcriptome data.

Data sheet shows **a)** metabolism-related genes upregulated in SDH-mutated PCC/PGL while **b)** metabolism-related genes downregulated in SDH-mutated PCC/PGL. Data sheet **c)** Gene ontology analysis of differentially regulated pathways in SDH-mutated PCC/PGL and the corresponding schematic illustration is shown in **d).** Data sheet **e)** shows the expression of the same genes in mouse imCC and MAF, knockout for Sdhb or wild-type (WT). SPO:sporadic.

Supplementary Table 2- Immunohistochemical evaluation of PC and SLC25A13 levels in human PGL/PCC.

			IHC score	
**Tumour ID**	**Mutation**	**Tumour site**	**PC**	**SLC25A13**
CIT_200	*SDHB*	LA	0.5	3
CIT_208	*SDHB*	Meta	1	2
CIT_209	*SDHB*	Meta	NA	2.5
PGL1	*SDHB*	Meta	NA	2
PGL2	*SDHB*	APG	2	2
CIT_201	*SDHB*	APG	1	3
PGL3	*SDHB*	RA	2	3
CIT_207	*SDHC*	TPG	1	2
CIT_158	*SDHD*	APG	1.5	3
PGL4	*SDHD*	CPG	1.5	2.5
CIT_048	*SDHD*	TPG	2	3
PGL5	*SDHD*	CPG	2	3
CIT_028	*SDHB*	LA	2	3
PGL6	*SDHD*	APG	2	3
PGL7	*SDHC*	PGL	1.5	2
PGL8	*SDHD*	CPG	NA	3
CIT_041	*RETs*	RA	0	0
CIT_044	*RET*	LA	0.5	2
CIT_086	*NF1*	LA	0.5	1
PGL9	*RET*	RA	0	0
CIT_070	*RETs*	LA	0.5	1
CIT_061	*NF1s*	LA	0	1
CIT_016	*NF1*	RA	0	2
CIT_115	*NF1*	LA	0.5	0
PGL10	*NF1s*	LA	0	1
CIT_034	*TMEM127*	RA	NA	1
CIT_133	*RET*	LA	0	1

LA: Left adrenal; RA: Right adrenal; TPG: Thoracic paraganglioma; CPG: Cervical paraganglioma; APG: Abdominal paraganglioma; PC: pyruvate carboxylase; IHC: immunohistochemistry
